# QAMaster: A new software framework for phantom‐based computed tomography quality assurance

**DOI:** 10.1002/acm2.13588

**Published:** 2022-03-17

**Authors:** Andre Karius, Christoph Bert

**Affiliations:** ^1^ Department of Radiation Oncology Universitätsklinikum Erlangen, Friedrich‐Alexander Universität Erlangen‐Nürnberg Erlangen Germany; ^2^ Comprehensive Cancer Center Erlangen‐EMN (CCC ER‐EMN) Erlangen Germany

**Keywords:** computed tomography, cone beam computed tomography, image quality assurance, phantom‐based software framework

## Abstract

The regular evaluation of imaging performance of computed tomography (CT) scanners is essential for CT quality assurance. For automation of this process, the software *QAMaster* was developed at the Universitätsklinikum Erlangen, which provides based on CT scans of the CatPhan® 504 (The Phantom Laboratory, Salem, USA) automated image quality analysis and documentation by evaluating CT number accuracy, spatial linearity, uniformity, contrast‐noise‐ratio, spatial resolution, noise, and slice thickness. Dose assessment is supported by calculations of the weighted computed tomography dose index (CTDI*_w_*) and weighted cone beam dose index (CBDI*_w_*).

*QAMaster* was tested with CatPhan® 504 scans and compared to manual evaluations of these scans, whereby high consistency of the respective results was observed. The CT numbers, spatial linearity, uniformity, contrast‐noise‐ratio, noise, and slice thickness deviated by only (0.13 ± 0.25) HU, (0.02 ± 0.05) mm, (−0.01 ± 0.03)%, 0.8 ± 1.8, (0.131 ± 0.05) HU, and (0.004 ± 0.005) mm between both evaluations, respectively. The *QAMaster* results for spatial resolution did not differ significantly (*p* = 0.34) from the CatPhan® 504 based manual resolution assessment. Dose computations were fully consistent between *QAMaster* and manual calculations. Thus, *QAMaster* proved to be a comprehensive and functional software for performing an automated CT quality assurance routine. *QAMaster* will be open‐source after its release.

## 1 INTRODUCTION

According to the as‐low‐as‐reasonable‐achievable‐(ALARA)‐principle,^1^ it must be ensured that the clinically required image quality of (cone beam) computed tomography (CBCT/CT) scans is accompanied by the lowest possible radiation exposure. A foundation for this is a regular quality assurance routine (QAR),^1^, ^2^ which evaluates a scanner's performance regarding image quality and dose. This is done using dedicated phantoms, for which automatization is preferable for securing reproducibility.

Some scientific publications^3^, ^4^ described automated image analyses based on self‐developed phantoms. These analyses allow comprehensive assessments of scans, but disadvantageously the developed phantoms are not commercially available or have to be purchased separately. Thus, using standard phantoms like the CatPhan® 504 (short: CatPhan; The Phantom Laboratory, Salem, USA) is preferable for QARs and represents the common practice in many medical facilities.

Currently, some tools, for example, myQA (IBA, Schwarzenbruck, Germany), exist which perform CatPhan‐based image analysis. These and similar products, however, often appear as black‐box tools, whose analysis procedures are not exactly known to the user and are also not alterable to be user‐specific. In many cases, such as with myQA, the offered QAR is only semi‐automated and requires manual adjustments of, for example, region of interest (ROI) positioning. This leads to both increased time requirements for the medical staff and a partly significant inter‐observer variability that may result in misleading QAR outcomes in clinical practice. Especially for changing/rotating staff, a QAR standardization by means of a simple “one‐click” solution is most desirable. At the same time, respective frameworks should provide for more experienced quality assurance (QA)‐executors the flexibility for easily and quickly integrating new QA checks into the workflow, and for modifying/adapting existing checks to (changing) research and QA requirements. A fully integrated documentation of the achieved QA results, also fully automated, is considered crucial for detecting gradual performance alterations of scanners by ensuring long‐term traceability of completed QA executions. The direct comparison of the obtained results to baselines and well‐established tolerance levels, such as, for example, provided by EFOMP,^2^ is very important in this respect as well. However, all these aspects are unfortunately not always covered by the currently available tools. Moreover, for both QA and research purposes, Fourier‐based metrics, such as particularly the noise power spectrum, represent fundamental quality metrics for the quantitative description of novel imaging modalities and systems,^3^ but are in general also not provided by the existing tools.

To overcome the described problems, we developed the software *QAMaster* that provides a fully automated CatPhan‐based imaging performance analysis with complete documentation by integrating common image parameters into one comprehensive framework. In addition to standard image quality parameters such as CT number accuracy or uniformity, the modulation transfer function (MTF) and noise power spectrum as Fourier‐based metrics are assessed and allow a more in‐depth and profound image quality analysis than currently available tools. *QAMaster* overcomes the black‐box problem, since its source code will be freely readable and thus modifiable individually, if desired. Thus, the supplied QAR can be customized to suit the particular requirements of individual hospitals. The presentation of *QAMaster* forms the scope of this work.

## 2 MATERIALS AND METHODS

### 2.1 CatPhan® 504

**FIGURE 1:**
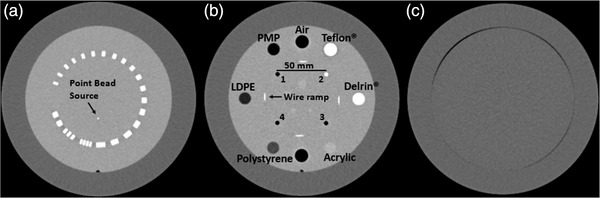
Computed tomography (CT)‐scans of the CatPhan sections CTP528 (a; used for MTF determination), CTP404 (b; contains inserts, rods and ramps for determining CT number accuracy, contrast‐noise‐ratio (CNR), spatial linearity, and reconstructed slice thickness) and CTP486 (c; used for evaluating uniformity and noise characteristics). The rods in CTP404 are labeled for distinctiveness with the numbers 1– 4. The non‐labeled insert at 6 o'clock position in (b) represents an additional, second air insert

The CatPhan is a modular phantom consisting of four individual sections, for which a detailed description is given by the manufacturer.^5^ *QAMaster* considers for image analysis the sections CTP404 (for evaluating CT number accuracy, contrast‐noise‐ratio (CNR), spatial linearity, and slice thickness), CTP528 (spatial resolution), and the water‐like^5^ CTP486 (uniformity, noise). Details regarding the modules’ structure are provided in Figure 1.

### 2.2 *QAMaster*: Internal structure

*QAMaster* is based on the programming language Python (v3.7.6) and Tkinter (v8.6.). After starting the software, the user reads in a CT/CBCT scan of the CatPhan via an implementation of The Visualization Toolkit (v9.0.1; Kitware, New York, USA). Several CT/CBCT systems and/or scan protocols, for which the QAR is to be performed or which shall be examined within the QAR, can be stored permanently in *QAMaster*. The user selects one of these created systems (or creates a new one) and enters the corresponding main menu. This allows to perform the QAR specifically adapted to different scanners/protocols and to separate the respective results systematically between them.

**FIGURE 2:**
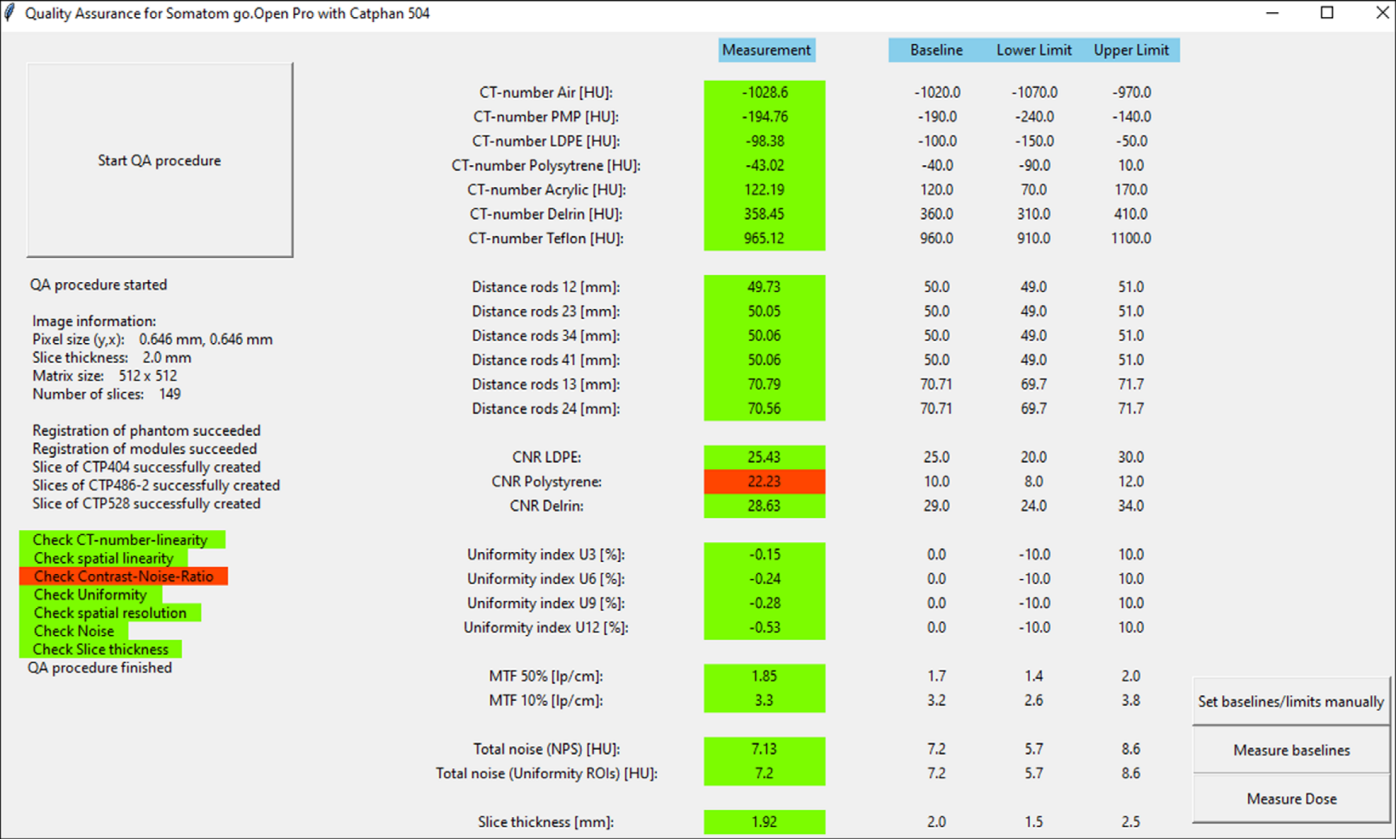
Main menu (after performing the quality assurance routine, QAR) of a computed tomography (CT) system in *QAMaster*, which gives a color‐coded overview of measured image parameters, baselines, and tolerances

The image parameters evaluated by *QAMaster* (Section 2.3) are displayed in the main menu (Figure 2) together with baselines, lower and upper tolerances respectively stored for the selected system. All baselines and tolerances can be manually changed or measured automatically. In the automated measurement, the individual image parameters are calculated as described below and set as baselines. The corresponding tolerances are then determined and pre‐configured for each parameter as shown in Table 1 based on recommendations of EFOMP^2^ and DIN 61223‐2‐6^6^ for CBCT and CT QA, respectively.

**TABLE 1:** Setting of tolerances by *QAMaster* considering the measured baselines. The tolerances were chosen based on the regulations given by EFOMP^2^ and the DIN 61223‐2‐6^6^ for QA in CBCT and CT, respectively

**Parameter**	**Tolerances: baseline** ±
CT numbers	50 HU
Distances between rods	1 mm
Slice thickness	0.5 mm
Uniformity indices	10%
HWHM/HWTM of MTF, CNRs, noise	20%

Abbreviations: CNR, contrast‐noise‐ratio; CT, computed tomography; HWHM, half width half maximum; HWTM, half width tenth maximum; MTF, modulation transfer function; QA, quality assurance.

Within the main menu the QAR can be started. The software automatically detects threshold‐based all outer margins/edges of the CatPhan on the acquired images. Based on these detections and the known phantom structure,^5^ *QAMaster* subsequently identifies the required (see Section 2.3) slices of the CTP404 and CTP486, as well as the point source of the CTP528. Since the mentioned margin detections are performed slice wise, no translation or tilt corrections are required for a proper operation of *QAMaster*. The positioning of the CatPhan on its case^5^ also eliminates the need for rotation corrections. Based on the detections, *QAMaster* calculates the image parameters described in Section 2.3. The results are stored on the user‐PC and displayed each marked with a green/red label if they lie/lie not within the respective tolerances.

### 2.3 *QAMaster*: Quality parameters

For evaluating **CT number accuracy**, *QAMaster* detects on the central slice of the CTP404 the individual inserts and centers a circular ROI within each. The radii of the ROIs are 1 mm smaller than the radii of the inserts, to account for CT number fluctuations at the insert edges. The CT number of an insert is calculated as mean pixel value of the respective ROI.

To evaluate **spatial linearity** indicating the image fidelity of scans, *QAMaster* determines the centers of the Teflon® and the three air rods by threshold‐based edge detection. The four sides and two diagonal lengths of the quadrilateral spanned by the rods are measured as the Euclidian distance between the individual rod centers.

**FIGURE 3:**
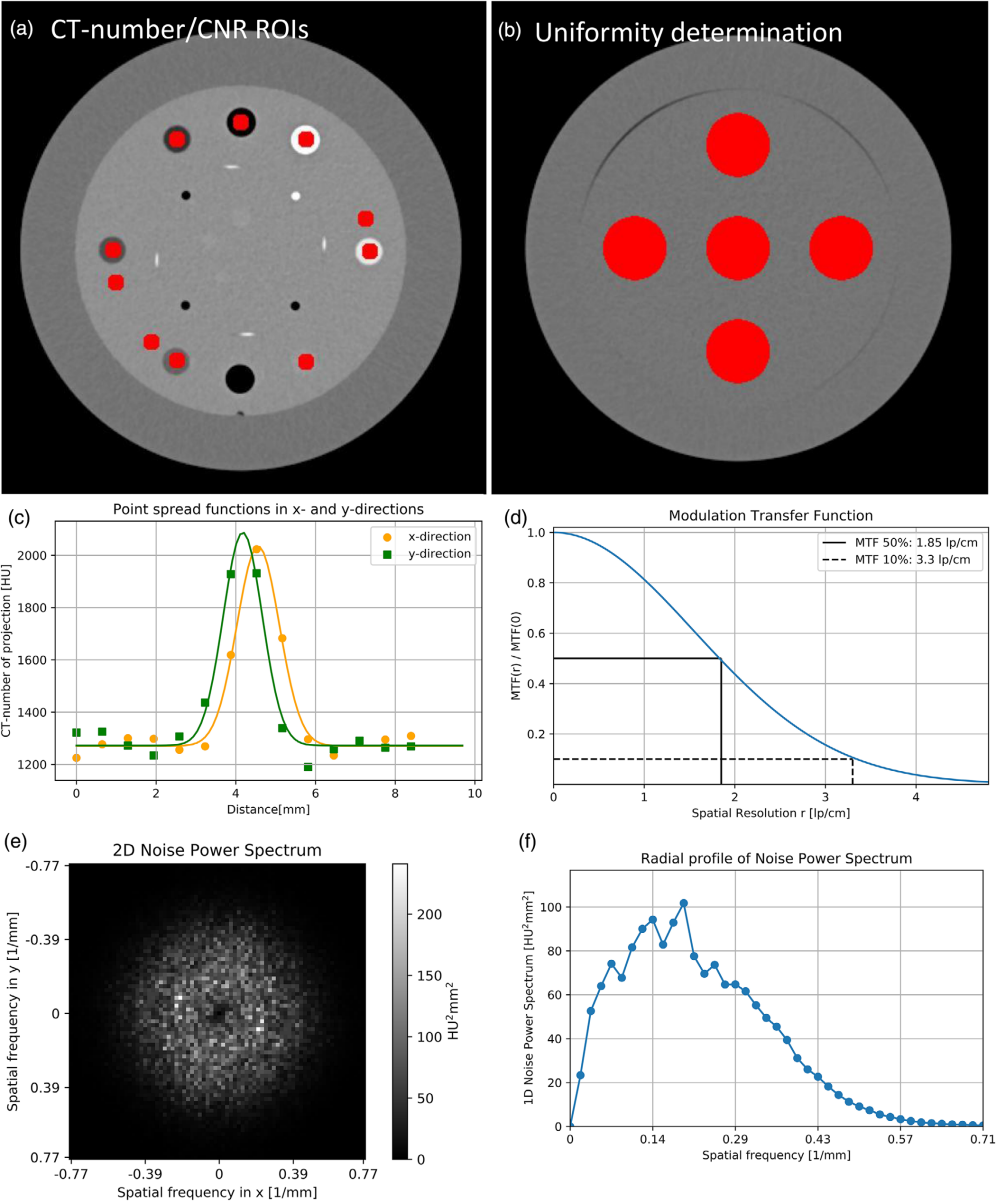
Graphical output by *QAMaster*: Shown are the regions of interest (ROIs) for determining contrast‐noise‐ratio (CNR), computed tomography (CT) number accuracy (both a) and uniformity (b), the calculated point‐spread functions (c) and MTF (d) as well as the 2D (e) and 1D (f) noise‐power spectrum (NPS)

**Image** **u** **niformity** is determined using the central slice of the CTP486 by the mean CT number of a ROI centered within the slice and of additional ROIs each placed peripherally at *i*‐clock position (i∈{3,6,9,12})^2^ with a distance of 1 cm from the module margin (see Figure 3b). All ROIs have a radius of 1.5 cm corresponding to 20%^2^ of the module radius. For each peripheral ROI, the uniformity index^7^ is calculated as follows:

(1) $$U_{i}=\frac{\mathrm{RO}I_{i}-\mathrm{RO}I_{\mathrm{central}}}{\mathrm{RO}I_{\mathrm{central}}+100}\;\;\cdot 100\%\;.$$

The addition of 100 is different to the original approach of Kyriakou et al.,^7^ where a value of 1000 was added in the denominator. As a result of our approach, the uniformity indices provide, with a mean CT number of the central ROI of about zero, a direct approximation of the absolute CT number deviations between peripheral and central regions.

**CNR** is calculated based on the central CTP404 slice for the LDPE (similar to fat), polystyrene (soft tissue), and Delrin® (bone) insert as proposed by Sheth et al.:^8^

(2) $$\mathrm{CN}R_{\mathrm{insert}}=\frac{\left|\mathrm{RO}I_{\mathrm{bkg}}-\mathrm{RO}I_{\mathrm{insert}}\right|}{\sqrt{\frac{1}{2}\cdot \left({\sigma_{\mathrm{bkg}}}^{2}+{\sigma_{\mathrm{insert}}}^{2}\right)}}\;\;.$$

$\mathrm{RO}I_{\mathrm{insert}\;}$represents the mean value of the respective insert determined as above and $\sigma_{\mathrm{insert}\;}$the associated CT number standard deviation. $\mathrm{RO}I_{\mathrm{bkg}\;\;}$and $\sigma_{\mathrm{bkg}\;}$denote the CT number mean and standard deviation of a background ROI adjacent to the respective insert. The background ROIs have the same size and are placed slightly rotated, but equally distant from the CTP404 center as the insert ROIs (see Figure 3a). The denominator in Equation (2) calculates the noise as pooled variance^8^ out of the ROIs’ standard deviations.

****Spatial resolution**** is evaluated considering a 5 × 5 mm^2^ ROI centered above the detected point source of CTP528. The CT numbers of the ROI's pixel are summed both row‐ and column‐wise, thus yielding one so‐called CT number projection for each of both image dimensions *x* and *y*. Both projections are fitted with a Gaussian distribution (see Figure 3c), which are shifted to a common center and averaged to obtain the radial point‐spread function, PSF(r) ($r\;=\sqrt{x^{2}+y^{2}\;}\;)$. Hankel‐transformation yields the modulation transfer function MTF($f_{r}$)^1^:

$$\mathrm{MTF}\;\left(f_{r}\right)=\;\int_{0}^{\infty }2\pi r\cdot \mathrm{PSF}\left(r\right)\cdot \;J_{0}\left(2\pi f_{r}\;\cdot r\right)\;dr\;.$$

$J_{0\;}\;$denotes the first‐type, zero‐order Bessel function. *QAMaster* outputs the MTF($f_{r}$) normalized to MTF(0) graphically (Figure 3d) and tabulated. In addition, the spatial frequencies $f_{r}\;$of the half width half maximum (HWHM) and half width tenth maximum (HWTM) of the MTF are determined for checking against respective tolerances.

**Noise** is characterized by calculating a noise‐power spectrum (NPS) based on a difference image of the central CTP486 slice and a slice spaced for times the slice thickness. On the difference image, the number $N_{\mathrm{ROI}}$ of about 200 ROIs of size 5 × 5 cm^2^, whose centers have a distance of ≤4 cm to the CTP486 center, yield the two‐dimensional (2D) NPS as function of the spatial frequencies $f_{x}$ and $f_{y}\;$as proposed by Steiding et al.:^3^

(3) $$\mathrm{NPS}\;\left(f_{x},\;f_{y}\right)=\;\frac{1}{N_{\mathrm{ROI}}}\cdot \frac{\Delta x\Delta y}{N_{x}N_{y}}\cdot \sum_{i\;=\;1}^{N_{\mathrm{ROI}}}\frac{|\mathrm{DFT}\left\{\mathrm{RO}I_{i}-CT_{i}\right\}{|}^{2}}{2}\;.$$

Δx, Δy are the pixel dimensions and *N_x_*, *N_y_* the number of pixels of each ROI in the *x*‐ and *y*‐direction, respectively. All ROIs, ROI*_i_*_,_ are offset‐corrected with their respective mean CT number, CT*_i_*_,_ and discretely Fourier transformed (DFT). The factor $\frac{1}{\;2}$ considers the noise increase associated with the difference image approach.^3^ The 2D NPS is graphically output (Figure 3e). Additionally, a 1D NPS(f_r_) (with $f_{r}=\;\sqrt{{f_{x}}^{2}+{f_{y}}^{2}}$) as averaged radial profile of the 2D NPS is determined (Figure 3f) and output both graphically and tabulated. The square root of its discrete integral yields a scalar measure $\Upsilon_{\mathrm{NPS}}$ for the image noise:

(4) $$\Upsilon_{\mathrm{NPS}}=\;\sqrt{2\pi \;\cdot \;\sum_{\;f_{r}=\;0}^{f_{r,\;\max}}\mathrm{NPS}\left(f_{r}\right)\cdot f_{r}\cdot \Delta f_{r}}\;.\;$$

$\Delta f_{r}\;$represent the spectral sampling distance^3^ of the 1D NPS and *f*_r,max_ its maximum frequency.

For capturing also systematic CT number fluctuations, *QAMaster* additionally calculates image noise $\Upsilon_{\mathrm{ROIs}}\;$as mean of the CT number standard deviations $\sigma_{i}$ of the five ROIs created to evaluate uniformity (see above):

(5) $$\Upsilon_{\mathrm{ROIs}}=\;\frac{1}{5}\cdot \sum_{i\;=\;1}^{5}\sigma_{i}\;.\;$$

The **reconstructed slice thickness** is evaluated by determining the CT number profile of a CTP404 ramp, whose full width half maximum (FWHM) is calculated across the module's three central slices. Due to the geometric arrangement of the ramps, the slice thickness is obtained by multiplication of the FWHM with the tangent of their inclination angle^5^ and division by 3, the number of considered slices.

****Dose calculations**** can be performed based on measurements using an IEC 61223‐2‐6 compliant dosimetry phantom^6^ and 100 mm pencil ionization chamber. As input the dose length products (DLPs) measured in the central and four peripheral boreholes of the phantom are required. *QAMaster* calculates the weighted computed tomography dose index (CTDI*_w_*)^1^ or weighted cone beam dose index (CBDI*_w_*)^9^:

(6) $$\mathrm{CTD}I_{w}=\frac{\mathrm{DL}P_{\mathrm{central}}}{3C}\;+\;\frac{2\cdot \;\left(\mathrm{DL}P_{\mathrm{peripher}}\right)}{3C}$$

(7) $$\mathrm{CBD}I_{w}=\frac{\mathrm{DL}P_{\mathrm{central}}}{300\;\mathrm{mm}}\;+\;\frac{2\cdot \;\left(\mathrm{DL}P_{\mathrm{peripher}}\right)}{300\;\mathrm{mm}}.$$

(DLP_peripher_) is the mean of the four DLPs measured in the peripheral positions and *C* is the user‐insertable nominal beam width of the investigated scanner.

### 2.4 Validation of *QAMaster*

For the validation of *QAMaster*, the results obtained with the software were compared to manual calculations of the image parameters described in Section 2.3 based on ROIs manually created within the RadiAnt™ DICOM Viewer (v2020.1.1; Medixant, Poznan, Poland). The latter method is called Human Observer (HO). *QAMaster*’s dose calculations were verified by generating 100 random combinations of 5 DLPs (one DLP for each borehole of the dosimetry phantom) and comparing the CTDI*_w_* and CBDI*_w_* calculations by the software to calculations by hand. HO‐based evaluation of spatial resolution was twofold: (i) the MTF was obtained by spatial derivation and subsequent DFT of the oversampled edge spread function^10^ (OESF) of an unresolvable line‐pair structure of the CTP528 slightly tilted within the axial plane. (ii) The MTF was also determined by the DFT of an oversampled line spread function^11^ (OLSF) created by the scan of a 60 μm thick metal wire, that was spanned with an angle of 10° in the coronal plane against the scanner's rotational axis. Noise was determined as HO only by using the uniformity‐ROI method and Equation (5).

To verify the correct identification and detection of the individual phantom structures needed by *QAMaste*r for image analysis (Section 2.3), the CatPhan was scanned with a SOMATOM go.Open Pro (Siemens, Erlangen, Germany) using 15 different protocols. The protocols (listed in the supporting information) had varying combinations of kernels (smooth to medium sharp), pixel sizes (0.4 × 0.4 mm^2^ to 0.7 × 0.7 mm^2^, 512 × 512 matrix), slice thickness (0.6–5 mm), and dose levels (CTDI*_w_* from 3.4 to 6.8 mGy). The results obtained with *QAMaster* were compared to the HO results to check the software's functionality. This comparison was performed for four scans of the CatPhan recorded with the scan parameters: 120 kV, 90 mAs_eff_, matrix 512 × 512, voxel size 0.5 × 0.5 × 3 mm^3^, smooth kernel.

Deviations between QAMaster and HO were tested with a two‐sided two‐sample *t*‐tests at a significance level, α=5%.

## 3 RESULTS

*QAMaster* reliably detected the inserts, rods, and ramps of the CTP404, the point source of the CTP528, and the two CTP486 slices required for a proper operation of the software and a complete image analysis in all cases for each of the 15 used protocols. The ROIs for evaluating CT number accuracy, CNR, uniformity, and noise were correctly created on all scans. No false detections or misplaced ROIs were observed. The values of CTDI*_w_* and CBDI*_w_* calculated by *QAMaster* were identical to the manual calculations for all tested DLP‐combinations, as summarized in the supporting information.

The mean deviation between the distances of the CTP404 rods calculated with *QAMaster* and as HO was (0.02 ± 0.05) mm, between the reconstructed slice thickness (0.004 ± 0.005) mm and between the uniformity indices (−0.01 ± 0.03)%. The CT numbers of the individual inserts differed on average by (0.13± 0.25) HU, the CNRs by 0.8 ± 1.8. All deviations were not statistically significant and were negligibly small compared to the absolute values of each of the parameters.

**FIGURE 4:**
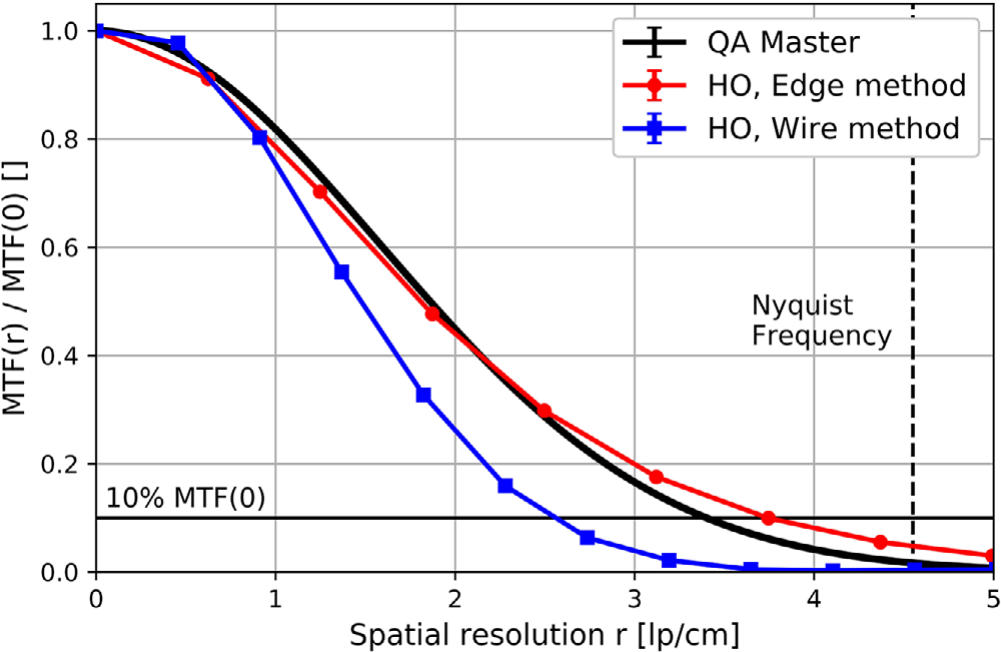
Comparison of the modulation transfer functions (MTFs) calculated by *QAMaster* and with both human observer (HO) methods

The MTFs calculated with *QAMaster* and as HO using the OESF are shown in Figure 4 and did not differ significantly (*p* = 0.34). However, the MTF determined as HO using the OLSF of the scanned wire deviated clearly from the other two generated MTFs.

A 2D and 1D NPS of the performed scans output by *QAMaster* are illustrated in Figure 3e,f. The image noise determined via both *QAMaster* methods and as HO differed by a maximum of (0.131 ± 0.05) HU. This difference was attributed to the not exactly reproducible positioning of the uniformity‐ROIs as HO as well as systematic noise on the scans not accounted for with the difference‐image approach used for NPS calculations.

## 4 DISCUSSION AND CONCLUSION

Between *QAMaster* and HO high levels of consistency and conformity of the calculated image parameters and dose metrics w found. Except of in the evaluation of the MTF, no systematic and statistically significant deviations between both methods were observed. The software detected the CatPhan structures needed for image analysis for all investigated scan protocols. *QAMaster* therefore proved to be a comprehensive, functional, and reliable software for performing an automated CT QAR for this scan protocols.

A weak point of *QAMaster* is in the evaluation of the spatial resolution. The occurrence of artifacts or strong noise in the scans in the vicinity of the CTP528 point source can lead to uncertainties in fitting single CT number projections (Figure 3c) and thus to incorrect calculations of the MTF. This behavior was particularly observed in Figure 4, and attributed to shadow artifacts in the immediate vicinity of the strongly attenuating point source as well as the line‐pair structures, which led to decreases of the respective CT numbers. These artifact‐related CT number reductions influenced the CT number profile of the OESF as well as the CT number projections fitted by *QAMaster* and led to a stretching of the corresponding MTFs compared to the evaluation of the resolution using the tensioned wire. The fact that the artifacts affected point‐source and line‐pair structures equally explains the agreement of the MTFs determined with both methods.

Although the exact determination of the MTF is of high importance for the characterization of the spatial resolution of a CT system, it is of secondary relevance for QA purposes, where mainly the reproducibility of the image quality and thus of the MTF is essential. In this respect, *QAMaster* is able to fulfill its purpose. Nevertheless, the limitations of MTF determination using the point source of the CTP528 has to be investigated in further studies, also using other CT systems. In this context, it has to be taken into account that the evaluation of the MTF via a point source is generally not as robust and stable^11^ as, for example, the performed HO methods. However, the point source appeared as the only way for calculating the MTF using the CatPhan, since a determination via the line‐pair structures of the CTP528 was not appropriate due to strong image artifacts within them, which significantly influenced the corresponding modulation.

In summary, *QAMaster* provides a very comprehensive, fully automated image quality analysis of CatPhan scans with complete documentation of the results and access to the source code. This distinguishes the software from already available tools such as myQA, for which image quality analyses are partly only semi‐automated and require manual user‐intervention, for example, for controlling the exact positioning of ROIs. The latter results in both increased time requirements for the executing staff and a strong inter‐observer variability, which may lead to misleading QA outcomes. Therefore, *QAMaster*’s full automatization is highly preferred. The complete documentation of all *QAMaster* results on the user's PC particularly enables their long‐term traceability and thus also the identification of even gradual scanner performance variations. Moreover, the already available programs represent in many cases black‐box tools, whose analysis methods/calculation procedures are not exactly known to the user. Hence, it is in general not possible to adapt the QAR to the needs of the respective hospital, for example, by deleting individual QA checks, implementing additional checks, or modifying/adapting checks. *QAMaster* overcomes this problem, since its source code will be freely readable and thus all calculation procedures are known in detail. Thus, if desired, the user can delete or modify QA checks already existing in *QAMaster*, or implement additional checks directly into the provided framework. This, combined with the full automatization of *QAMaster*, allows the software to be used not only for QA, but in particular also for research purposes. The user‐adaption of image quality analyses to respective research questions using the *QAMaster* automatization and documentation is explicitly supported and foreseen by its developers. For example, the implementation of an automated analysis of hundreds of scans in direct succession with respective documentation of the results is feasible in principle.

The calculation of Fourier‐based metrics, such as MTF and in particular NPS, by *QAMaster* exceeds the capabilities of existing tools such as myQA. However, these Fourier‐based metrics are fundamental for the quantitative description of CT/CBCT scans,^3^ as they allow for an in‐depth characterization of the imaging performance of scanners. In particular, for CT research like the development/optimization/assessment of reconstruction algorithms in both clinical and industrial environments, the considerations of MTF and NPS are crucial. *QAMaster* can strongly support in this respect by providing a well‐founded analysis of CatPhan scans. To our knowledge, there is currently no other tool on the free market that allows as many image quality checks at the same time as *QAMaster* and thus analyzes the image quality of CT scans so comprehensively. All QA results are thereby directly checked against respective baselines and tolerances and thus a direct indication for the necessity of taking respective measures (e.g., scanner calibration) is provided. The full documentation and output of the results also allow their use for more advanced investigations in CT research beyond the interest of QA measures, such as, for example, for the calculation of further imaging parameters like the noise equivalent quanta out of MTF and NPS. Therefore, *QAMaster* can significantly assist and support in both research and, of course, the CT/CBCT QA performed by clinical medical physicists.

One drawback of *QAMaster* is that, as an in‐house development, it features currently no food and drug administration (FDA)‐approval or similar. However, open‐source possibilities for this are reviewed. Furthermore, unlike, for example, myQA, *QAMaster* does currently not feature a schedule function that actively reminds the clinical user to perform open QA measures. In this respect, we have made the experience that such a scheduling is often also performed by using either public calendars (e.g., Google Calendar; Google, Mountain View, USA) or calendar systems already existing within the workflow of the respective hospital. In addition, *QAMaster* is currently limited to the CatPhan only. This, however, we do not judge as major drawback compared to other software tools, since these are in many cases also limited to a specific phantom series, but of course as a drawback for the clinical user. For this reason, we aim at extending the software in future versions to phantoms other than the CatPhan, in order to broaden *QAMaster*’s clinical application possibilities. In particular, the extension of *QAMaster* to the QA of dual‐energy as well as four‐dimensional CT (4D‐CT) imaging will be targeted.

## CONFLICT OF INTEREST

The authors declare that there is no conflict of interest that could be perceived as prejudicing the impartiality of the research reported.

## AUTHOR CONTRIBUTIONS

Andre Karius developed the concept as well as methodology of the presented software and validated its functionality under extensive and constant exchange with Christoph Bert. Both revised the work critically, approved the final version to be published and are accountable for all aspects of the present work.

## Supporting information

Supporting Information

Supporting Information
