# Supplementary material for: QAMaster: A new software framework for phantom‐based computed tomography quality assurance
Source: J Appl Clin Med Phys. 2022 Mar 17;23(4):e13588. doi: 10.1002/acm2.13588 (PMC8992959; doi:10.1002/acm2.13588)
Supplement: Supplementary file 2 — Supporting Information [file ACM2-23-e13588-s002.pdf]

Supplementary File to QAMaster: A new software framework for phantom based CT quality assurance

In the following we present the scan protocols used to validate the software *QAMaster*. All scans were acquired with the conventional CT system SOMATOM go.Open Pro (Siemens Healthineers, Erlangen, Germany) and had a 512x512 matrix:

**No.**	**Tube voltage**	**Effective current-time-product**	**CTDI**	**Slice thickness**	**Pixel Size**	**Kernel**
#1	120 kV	90 mAs	6.2 mGy	0.6 mm	0.6x0.6 mm^2	Br40 (smooth)
#2	120 kV	90 mAs	6.2 mGy	0.8 mm	0.6x0.6 mm^2	Br40
#3	120 kV	90 mAs	6.2 mGy	1 mm	0.6x0.6 mm^2	Br40
#4	120 kV	90 mAs	6.2 mGy	2 mm	0.6x0.6 mm^2	Br40
#5	120 kV	90 mAs	6.2 mGy	2 mm	0.4x0.4 mm^2	Br40
#6	120 kV	90 mAs	6.2 mGy	2 mm	0.7x0.7 mm^2	Br40
#7	120 kV	90 mAs	6.2 mGy	3 mm	0.5x0.5 mm^2	Br40
#8	120 kV	90 mAs	6.2 mGy	3 mm	0.6x0.6 mm^2	Br40
#9	120 kV	90 mAs	6.2 mGy	4 mm	0.6x0.6 mm^2	Br40
#10	120 kV	90 mAs	6.2 mGy	5 mm	0.6x0.6 mm^2	Br40
#11	120 kV	90 mAs	6.2 mGy	2 mm	0.6x0.6 mm^2	Br36 (smooth)
#12	120 kV	90 mAs	6.2 mGy	2 mm	0.6x0.6 mm^2	Br44 (medium)
#13	120 kV	80 mAs	5.5 mGy	2 mm	0.6x0.6 mm^2	Br48 (medium-sharp)
#14	100 kV	80 mAs	3.4 mGy	2 mm	0.6x0.6 mm^2	Br40
#15	130 kV	80 mAs	6.8 mGy	2 mm	0.6x0.6 mm^2	Br40
